# Structure and Properties of Polysulfone Filled with Modified Twill Weave Carbon Fabrics

**DOI:** 10.3390/polym12010050

**Published:** 2019-12-30

**Authors:** Dilyus I. Chukov, Sarvarkhodza G. Nematulloev, Viсtor V. Tсherdyntsev, Valerii G. Torokhov, Andrey A. Stepashkin, Mikhail Y. Zadorozhnyy, Dmitry D. Zherebtsov, Galal Sherif

**Affiliations:** NUST “MISiS”, Center of composite materials, Leninskiy pr. 4, Moscow 119049, Russia; nematulloev.sarvar@yandex.ru (S.G.N.); vvch@misis.ru (V.V.T.); vgtorohov@gmail.com (V.G.T.); a.stepashkin@yandex.ru (A.A.S.); priboy38@mail.ru (M.Y.Z.); dmitry_zherebtsov@bk.ru (D.D.Z.); eng_galal_emad@mu.edu.eg (G.S.)

**Keywords:** polymer-matrix composites, carbon fibers, polysulfone, surface modification

## Abstract

Carbon fabrics are widely used in polymer based composites. Nowadays, most of the advanced high-performance composites are based on thermosetting polymer matrices such as epoxy resin. Thermoplastics have received high attention as polymer matrices due to their low curing duration, high chemical resistance, high recyclability, and mass production capability in comparison with thermosetting polymers. In this paper, we suggest thermoplastic based composite materials reinforced with carbon fibers. Composites based on polysulfone reinforced with carbon fabrics using polymer solvent impregnation were studied. It is well known that despite the excellent mechanical properties, carbon fibers possess poor wettability and adhesion to polymers because of the fiber surface chemical inertness and smoothness. Therefore, to improve the fiber–matrix interfacial interaction, the surface modification of the carbon fibers by thermal oxidation was used. It was shown that the surface modification resulted in a noticeable change in the functional composition of the carbon fibers’ surface and increased the mechanical properties of the polysulfone based composites. Significant increase in composites mechanical properties and thermal stability as a result of carbon fiber surface modification was observed.

## 1. Introduction

Among the various types of inorganic and organic fibers, carbon fibers (CFs) show the highest tensile strength and elastic modulus at relatively low density [1,2]. This is why CFs, despite their high cost, are widely investigated and applied. The most promising CF application is as a reinforcement for polymer based composites [1,2,3]. CF reinforced polymer composites have been widely used as engineering materials in the aeronautic and automotive industries. Specific applications of these composites include body structure for electric vehicles [4], wet clutches to distribute torque in vehicle drive-trains of automatic transmissions and limited slip differentials [5], deployable space structures [6], heat exchangers [7], electromagnetic shielding [8], ballistic protection [2,9], and some other engineering areas. For example, in [10,11,12], a family of novel high-strength, lightweight structural epoxy/CF composites with self-healing function were proposed for potential use in aerospace and aeronautical structures, sports utilities, etc.

The properties of CF reinforced polymer composites strongly depend on the fiber shape and location in the matrix. Polymer composites filled with short CFs are widely used as materials with good mechanical and tribological performance [3,13,14,15]. The technology of the formation of such composites is relatively cheap and easy [16,17], moreover, they are suitable for production by additive manufacturing [18]. Short CFs can either be chaotically distributed in a polymer matrix or oriented; in the latter, composites show anisotropy in mechanical [18], thermal [18], and tribological [19] properties. However, short CF filled polymers do not achieve the mechanical characteristics required for high-performance structural application in the aeronautic and automotive industries. Realization of the excellent mechanical properties of CFs in the polymer matrix can be more effectively achieved by using continuous fibers as reinforcement.

Continuous CF reinforced unidirectional composites are popular objects for modeling and theoretical approaches [20,21]. Unidirectional composites allow for the realization of CF mechanical characteristics better than 2D ones [1]. Unidirectional composites based on thermoplastics can be easily produced by simply using impregnation with polymer melt [22]. Application of unidirectional composites is restricted to very specific areas [6], however, such composites are promising as a semi-product in advanced additive manufacturing [23].

Most of the investigation and structural applications of CF reinforced polymer composites is based on 2D CF fabrics. Among the carbon fabrics, the most commonly used as reinforcement in polymer composites are woven fabrics, whereas knitted and braided fabrics are used much less often [3]. The number of weave structures that can be produced is practically unlimited, but in the investigations and applications, based structures such as plain, twill and satin are most commonly used. The weave structure of reinforcing woven CF fabrics can significantly affect the mechanical behavior of polymer composites. For instance, a comparison of epoxy based composites reinforced with CF fabrics showed that composites with twill weave fabrics show a higher shear modulus, shear strength, and ultimate strength than composites reinforced with satin weave CFs [24]. Similar results were observed in [25], where tensile tests showed that epoxy based composites reinforced with twill weave CF fabrics possessed higher magnitudes of strength, modulus, and strain than composites containing satin weave CFs. Mechanical tests, carried out with epoxy based composites, showed that the tensile properties of composites reinforced with twill weave CFs were more stable with an increase in the strain rate than the properties of composites with plain and satin weave CF fabrics [26]. The investigation of polyetherimide based composites showed that composites containing twill weave CF fabrics showed better tensile and flexural properties than composites filled with plain and satin weave CFs; however, composites with twill weave CFs had good wear resistance properties in adhesive mode only, whereas in abrasive wear mode, better properties were observed for composites with satin weave CFs and in erosive wear mode both satin and plain weave CF filled composites shows better behavior than the twill weave reinforced one [27]. On the other hand, in [28], it was reported than epoxy composites filled with twill weave CFs showed lower tensile characteristics than composites reinforced with plain and satin weave CFs, both in static and dynamic test modes. Furthermore, twill weave CFs are often used as the model object in theoretical investigations [29,30] and can be a suitable material for the elaboration of new polymer based composites.

For critical structures operating under high loads and elevated temperatures, composite materials with matrices based on thermoplastic polymers are actively beginning to be applied. Thermoplastic based composites show a considerably higher static fracture toughness compared to the thermoset (epoxy) composites [3]. Important advantages of thermoplastics are their unlimited shelf life, low curing duration, maintainability (i.e., the ability to correct defects and damage by reheating), the possibility of reforming defective products [31], high environmental resistance, and high chemical resistance including to aviation fuels and oils [1].

In recent years, a number of thermoplastic based CF reinforced composites have been elaborated and investigated [32]. Among the thermoplastics, high performance polymers are of particular interest due to their thermal stability and high mechanical properties. Polysulfone (PSU) has one of the highest service temperatures of all melt-processable high performance polymers. The high temperature nature of the PSU allows them to be used in demanding applications that other polymeric materials cannot satisfy. PSU is highly resistant to acids, alkali, and electrolyte materials, oxidizing agents, surfactants, and hydrocarbon oils. Its resistance to high temperatures allows for the use of PSU as a flame retardant material [33].

PSU is frequently used as a modifier for the epoxy matrix for CF reinforced composites [34,35,36]. In the 1990s, CF reinforced PSU was widely considered to be promising in medicine for arthoplastic applications, where composites filled with short [37] or unidirectional [38,39,40] CFs were mainly used in these investigations. In recent years, only few papers related to PSU based CF reinforced composites have appeared. In [41], the laminated gradient PSU composites with the formation of layers by parallel laying of continuous CFs as materials for artificial intervertebral discs were elaborated. The authors in [42] report on the formation of thermal conductive short CF filled PSU composites by injection molding. No data on the PSU composites reinforced with CF fabrics were observed in the literature.

The aim of the present paper was to study the effect of CF surface modification by thermal oxidation on the mechanical and thermal properties of the polysulfone based composites reinforced with carbon fabrics. Whereas polymer melt impregnation, as was shown in [22], is a suitable method for PSU based unidirectional CF composites, in the case of the less permeable structure of CF fabrics, this technique is not effective. Recently, [43,44] suggested using a PSU solution impregnation method to obtain CF fabric reinforced PSU based composites. In the present study, we applied the PSU solution impregnation method to produce composites reinforced with CF twill weave fabrics.

## 2. Materials and Methods

### 2.1. Preparation of Carbon Fiber/Polysulfone Composites

**Figure 1:**
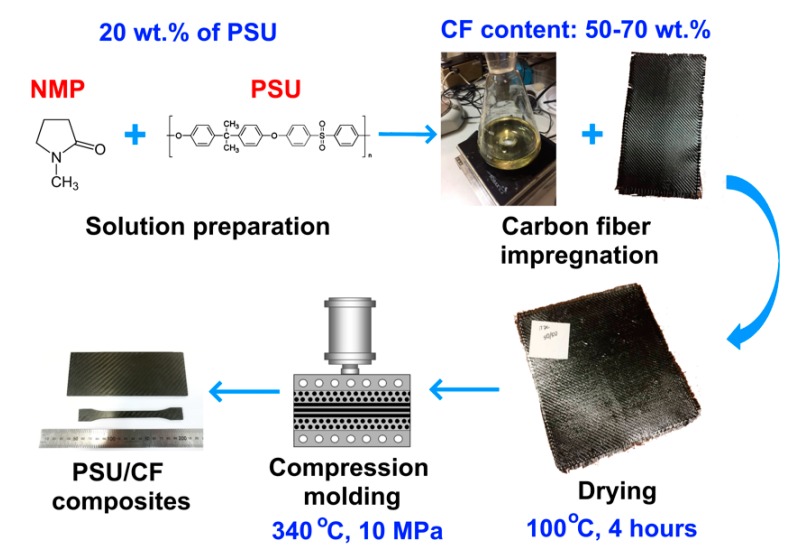
Scheme of the preparation of the carbon fiber reinforced polysulfone based composites.

Ultrason S2010 (BASF, Ludwigshafen, Germany) PSU and twill weave fabrics 3K-1200-200 (HC Composite, Moscow, Russia) based on high-modulus carbon fibers were used as the raw materials. Obtaining the composites was carried out in several stages including obtaining a polysulfone solution, impregnation of the CF with the solution; and drying, which resulted in prepregs (pre-impregnated fabrics). The prepregs were further cut into the desired shapes and molded by compression molding into the final composites. Figure 1 shows the scheme of the used process.

To obtain the PSU solutions, N-methyl-2-pyrrolidone (Eastchem, Jiangsu, China) was used as a solvent, and a 20 wt % PSU solution was formed using a magnetic stirrer. After impregnation, the carbon fabrics were dried at 100 °C. CF/PSU composites with various fiber to polymer ratios were produced: 50 wt % of the CF and 50 wt % of the PSU (denoted as 50/50); 60 wt % of the CF and 40 wt % of the PSU (60/40); and 70 wt % of the CF and 30 wt % of the PSU (70/30). These prepregs were stacked in the mold carefully to avoid misalignment. The stainless steel mold was previously coated with the mold release agent. During compression molding, the mold was heated to attain the temperature of 340 °C. The prepregs were compression molded at the above-mentioned temperature under the pressure of 10 MPa. Thereafter, samples were cooled under applied pressure. The specimens for investigation were cut from the composites in accordance with the standards for mechanical testing.

### 2.2. Carbon Fibers Surface Modification

To improve the fiber–matrix interfacial interaction, surface modification of the CF by thermal oxidation (TO) in an air atmosphere at 300, 400, and 500 °C was applied and the fibers denoted as TO 300, TO 400, and TO 500 °C, respectively. The muffle furnace was heated up to the desired temperature, then the fibers were loaded into the furnace and held for 30 min. The fiber to polymer ratio of 60/40 was used for all of the modified fiber reinforced composites.

### 2.3. Thermogravimetric Analysis

To study the kinetics of solvent removal, thermogravimetric analysis (TGA) was performed using the TA Instruments Q600 system (TA Instruments, New Castle, DE, USA). During the tests, the polymer solution was heated at a rate of 10 °C/min to various temperatures of isothermal exposure (80, 100, and 120 °C); after reaching the isotherm, air blowing was started. The total measurement time was 3 h. The weight of the samples of the studied materials was chosen as close as possible to each other and varied in the range of 20–25 mg.

### 2.4. Fourier-Transform Infrared Spectroscopy Analysis

The FTIR analysis was carried out using a Nicolet 380 spectrometer (Thermo Scientific, Waltham, MA, USA) with spectral range of 3750–650 cm^−1^ and resolution of 1 cm^−1^.

### 2.5. X-ray Photoelectron Spectroscopy

The x-ray photoelectron spectroscopy (XPS) studies of the CF were carried out using a ULVAC-PHI VersaProbe II spectrometer (ULVAC-PHI, Inc., Chigasaki, Kanagawa, Japan) with monochromatic Al Kα radiation (hν = 1486.6 eV), a power of 50 W, and a beam diameter of 200 μm. Atomic concentrations were determined from survey spectra using the method of relative elemental sensitivity factors. The binding energies (B.E.) of the photoelectron lines (C1s, O1s, N1s) were determined from high-resolution spectra taken at an analyzer transmittance energy of 23.5 eV and a data acquisition density of 0.2 eV/step. For decomposition of the photo peal, we used a Gaussian Lorentzian mix function and a linear background subtraction. XPS analysis of the modified fabrics was performed immediately after surface modification.

### 2.6. In-Plane Shear Strength Tests

In-plane shear strength of the composites was determined according to ASTM D 3846–02 using 80 × 10 × 4 mm samples at a crosshead speed of 1.3 mm/min. For the tests, a Zwick/Roell Z020 universal test machine (Zwick Roell Group, Ulm, Germany) was used. Compressive load was applied to a notched specimen of uniform width to measure the shear strength. The specimen was loaded edgewise in a supporting jig of the same description as that referenced in ASTM D 695 for testing thin specimens. A failure of the specimen occurs in shear between two centrally located notches machined halfway through its thickness and spaced a fixed distance apart on opposing faces. The distance between the notches was 6.5–8 mm.

### 2.7. Mechanical Tests

A Zwick/Roell Z020 universal test machine equipped with 1 and 20 kN sensors and a contact strain measurement system MultiXtens was used for mechanical tests. Tensile tests were performed in accordance with ISO 527:2009 for 110 × 10 × 2 mm samples and the flexural tests were performed in accordance with ISO 14125:1998 for 110 × 10 × 2 mm samples at a span length of 80 mm. Cross head speed during the tests was 10 mm/min. Five samples were tested for each type of composites in each test. Composite structure was investigated using a VEGA 3 TESCAN scanning electron microscope ((TESCAN ORSAY HOLDING, a.s., Brno–Kohoutovice, Czech Republic)) in a backscattered electron image mode. For the scanning electron microscopy (SEM) test, all specimens were sputter coated with a thin layer of carbon (10–15 nm) to provide the electrical conductivity of the samples.

### 2.8. Dynamic Mechanical Analysis

A Dynamic mechanical analyzer DMA Q800 (TA Instruments, New Castle, DE, USA) dynamic mechanical analyzer was used in these investigations. Specimens approximately 2 mm wide, 2 mm thick and 45 mm long were used for the DMA tests. The measurements were performed using a double cantilever clamp at a frequency of 1 Hz and deformation of 0.1%, in a temperature range from 30 to 220 °C and the heating rate was 2 °C/min.

## 3. Results and Discussion

### 3.1. Polysulfone Solution

**Figure 2:**
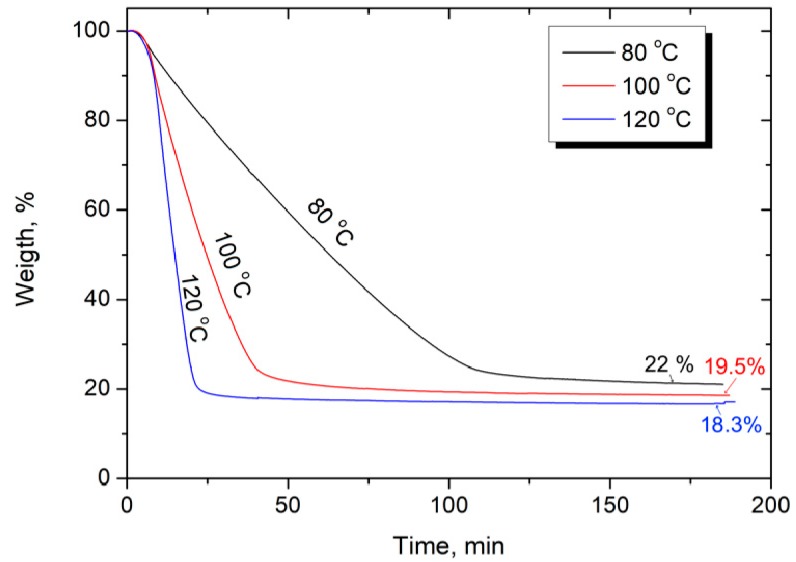
Solvent evaporation from Polysulfone/N-methyl-2-pyrrolidone solutions.

Due to the high melt viscosity of the main part of high-performance thermoplastic polymers, the forces exerted on the fibers during melt impregnation are extremely high and may cause fiber damage [45]. The viscosity of the polymer solution is much lower than that of the melt, which allows it to impregnate the fabric uniformly and increase the wettability of the fibers. Another important advantage is that, unlike the melt impregnation, solution impregnation can be carried out at room temperature, followed by removal of the solvent at elevated temperatures. Therefore, solution impregnation can be considered as the preferred method to prepare high performance composites based on a soluble high performance thermoplastic [46,47]. The main disadvantage of this approach is removing the solvent after impregnation. Incomplete removal may produce voids that have a deleterious effect on the mechanical properties of the composite. Obtaining composites with the best set of properties is possible only if the minimum amount of residual solvent is reached. The results of the solvent evaporation at various temperatures obtained by thermogravimetric analysis are presented in Figure 2. It was found that the kinetics of solvent removal strongly depend on the measurement temperature. The results show that the higher the temperature, the faster the TGA curve reaches a plateau, and if at 80 °C, intense mass loss (evaporation of the solvent) lasts about 110 min, then at 100 °C and 120 °C, the time to the plateau decreases to 50 and 25 min, respectively. It was found that drying at 80 °C did not completely remove the solvent, and its residual ratio was about 2 wt %, while, at higher temperatures, the solvent could be completely removed.

**Figure 3:**
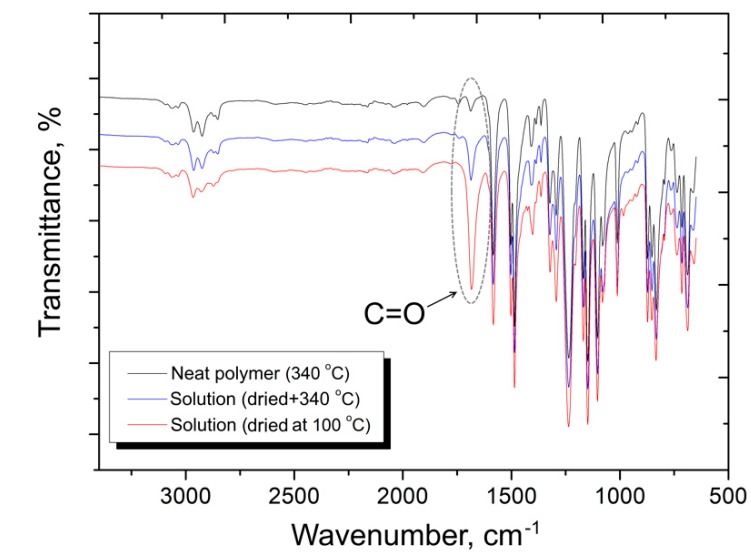
Infrared spectra of polysulfone after various treatments.

These results were further confirmed by FTIR spectroscopy of the PSU after various treatments. Figure 3 shows the FTIR spectra of pure PSU after compression molding at 340 °C (designated as the neat polymer (340 °C)); the polymer that was dissolved in N-methyl-2-pyrrolidone, dried at 100 °C, and compression molded at 340 °C (solution (dried at 340 °C)); and also the dissolved polymer just after drying at 100 °C (solution (dried at 100 °C)). Among the many observed peaks, a peak at 1690 cm^−1^ was chosen as the reference, which corresponds to C=O bond oscillations [48,49]. This peak can be associated with the presence of residual solvent in the sample or partial oxidation of the polymer during one or another treatment. It should be noted that during compression molding, there is no significant oxidation of the polymer, as evidenced by the low intensity of the peak at 1690 cm^−1^ for the pure PSU after compression molding at 340 °C, therefore in this case, we associated this peak with the presence of residual solvent. The FTIR results for the just dried PSU solution showed that the peak for the C=O bond oscillations had a rather high intensity, but after compression molding of the same sample, a noticeable decrease in the intensity of this peak occurred, which confirms the additional removal of the solvent during the compression molding. Therefore, it can be concluded that the residual solvent content in the final composites is very low and will not affect the composites’ mechanical and other performance properties. Any noticeable changes or appearance of new peaks in the FTIR spectra of the PSU samples after various treatments were not found.

### 3.2. Surface Modification of CFs

It is well known, that despite the excellent mechanical properties, CFs show poor wettability and adhesion to matrix polymers, most likely because the CF surface is chemically inert, smooth, and exhibits low surface free energy [50,51]. Thermal oxidation is one of the most widely used methods of CF surface modification, which allows for improvement in the fiber–matrix interaction in polymer based composites. We used various thermal oxidation temperatures (up to 500 °C) to study the effect of surface treatment on the fibers and composite structure and properties.

**Figure 4:**
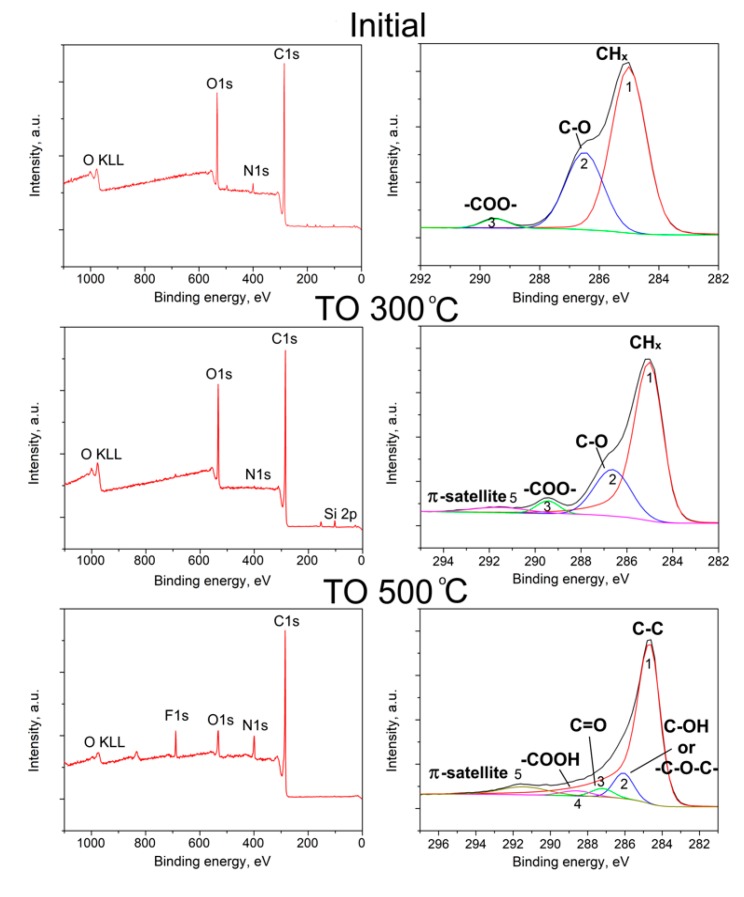
X-ray photoelectron spectroscopy survey spectra (left) and the high resolution spectra of C1s (right) of the initial and modified (TO 300 °C and TO 500 °C) carbon fibers.

To study the elemental composition and functional groups on the surface of the initial and modified carbon fibers, the x-ray photoelectron spectroscopy (XPS) method was used, which is the most commonly used method that provides comprehensive information about the CF surface. Elemental compositions and specific contents of various functional groups on the CF surface were investigated. The wide-scan spectra allows for the elemental compositions of the carbon fiber surface and the high resolution spectra of C1s peak to be obtained, which allows for the identification of functional groups on the CF surface, as shown in Figure 4 and the results are summarized in Table 1 and Table 2.

It was found that the used regimes of surface modifications resulted in a noticeable change in the elemental composition of the fiber surface. It is known that the sizing applied to the CF surface is usually an organic polymer such as polyetherimide, epoxy resin, etc., with a high oxygen content [52,53]. Additionally, amine hardeners are often used to cure epoxy resins. As a result, a sufficiently high content of oxygen and nitrogen was found in the initial fibers (18.0 and 3.0 at. %, respectively). The concentration of carbon on the surface of the initial fibers was 77.5 at. %. Surface modification of carbon fibers increased the concentration of carbon to 79.0 at. % and 82.5 at. % after thermal oxidation (TO) at 300 °C (denoted as TO 300 °C) and 500 °C (denoted as TO 300 °C), respectively. At the same time, if after thermal oxidation at a temperature of 300 °C the oxygen concentration did not significantly change, caused by incomplete removal of the sizing at this temperature, the modification at 500 °C decreased the oxygen concentration to 7.5 at. %. After treatment at 300 °C, the nitrogen concentration decreased, which is due to the partial removal of the sizing agent, while the clean surface of the fibers was depleted in nitrogen, which may be due to the fact that it is covered with a graphitized residue that is formed during the production of the fiber. After surface modification at 500 °C, the nitrogen content increased to 8.5%.

The high-resolution spectrum shown in Figure 4 (right) was used to analyze the C1s region. Each spectrum was decomposed into a set of subspectra that allowed for the detection of the specific contents of various functional groups on the CF surface. The initial CF surface C1s spectrum corresponds to the polymer spectrum, which is typical for sized CF. For the sample after treatment at 300 °C, it was found that there is no obvious asymmetry of the C1s spectrum, which means that removing the sizing from the CF was not completed in this treatment. Treatment at 500 °C resulted in a sharp change in the shape of the C1s spectrum and it acquired the typical peaks for clean CF surface features: the main asymmetric peak 1 is from graphitized carbon, and peaks 2, 3, and 4 correspond to functional groups.

The binding energies (E_B_) obtained from the C1s spectra are summarized in Table 2. Typical features of the C1s spectrum of the original fiber are the symmetric peak 1 (E_B_ = 285.0 eV) from CHx groups, peak 2 (E_B_ = 286.5 eV) from C–O bonds and peak 3 (E_B_ = 289.5 eV), which can be attributed to the N–COO– or –COO– group. The center of the oxygen spectrum is located at E_B_ = 532.8 eV, which can be attributed to several forms of oxygen. A high-resolution spectrum of nitrogen in the sample was not obtained; E_B_ was determined from a survey spectrum, 400.3 eV, and corresponded to the N–COO– group.

For the CF after treatment at 300 °C, the most satisfactory approximation of the C1s spectrum was made by adding a small asymmetry to the shape of peak 1, but the asymmetry parameters did not correspond to a pure carbon surface. Typical features of the C1s spectrum after this treatment are symmetric peak 1 (E_B_ = 285.0 eV) from the CHx groups, peak 2 (E_B_ = 286.5 eV) from the CO bonds, peak 3 (E_B_ = 289.5 eV) from the groups N–COO– or –COO–, and peak 5 (E_B_ = 291.5 eV)-π satellite [54,55]. TO 500 °C resulted in a significant change in the functional composition of the fibers, and several peaks related to the various functional groups were observed. The following peaks were detected in these spectra: peak 2 (E_B_ = 286.1 eV) from hydroxyl –C–OH, ethereal –C–O–C– or epoxy groups, and C–N– (it should be noted that the chemical shift for C–N is slightly less than C–O, however, it is almost impossible to distinguish them reliably), peak 3 (E_B_ = 287.5 eV) corresponded to double bonds C=O and –C=N, and peak 4 (E_B_ = 288.6 eV) carboxyl groups COOH– [56]. The presence of double bonds N=O and C=O was additionally confirmed by the O1s and N1s spectra, from which two components were distinguished. In the O1s spectrum, a peak at 531.3 eV corresponded to a double bond, and a peak at 533.4 eV to a single bond. Similarly, for the N1s spectrum, the peak at 398.7 eV corresponded to double bonds, and the peak at 400.5 eV to single bonds. Hence, the used regimes of modification allowed for a change in the CF surface chemical composition, and a greater number of functional groups on the surface appeared as a result of thermal oxidation. An increase in the activity of the CF surface affected the fiber–matrix interaction in the composites.

### 3.3. Interface Analysis of Carbon Fibers/Polysulfone Composites

**Figure 5:**
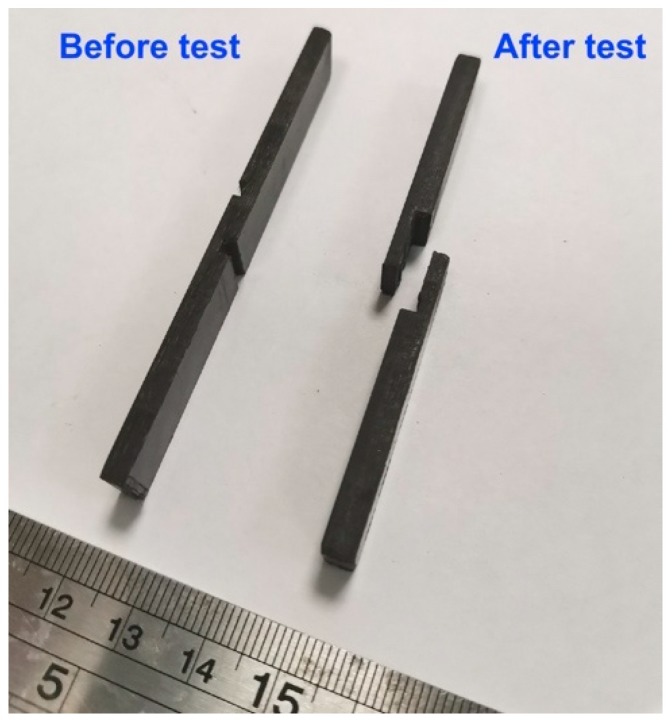
The experimental samples for the in-plane shear strength tests.

**Figure 6:**
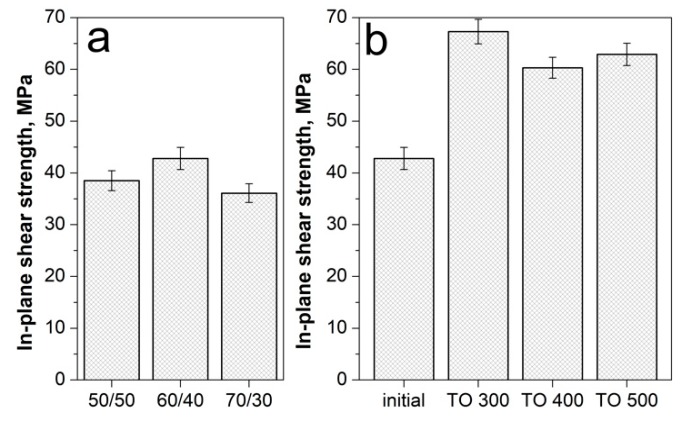
The in-plane shear strength of the CF/PSU composites depending on fiber content (**a**) and the fiber surface modification regime (**b**).

In-plane shear strength (IPSS) is an important indicator of the interfacial adhesion between the fiber and matrix [57,58]. Here, we used shear tests to evaluate the IPSS of the PSU based composites reinforced with the initial and surface modified carbon fibers. Figure 5 shows the outlook of the investigated samples before and after the shear test. It can be seen that destruction of samples proceeds along the layers of composites, thus the results of the shear strength tests can be unambiguously attributed to the fiber–matrix interfacial interaction. The results of the in-plane shear strength of the CF/PSU composites with various fiber to polymer ratios are shown in Figure 6a.

It was established that the shear strength of the composites with a 50/50 fiber to polymer ratio and reinforced with the initial fibers was 38.5 ± 1.4 MPa, for the 60/40 composites it was 42.8 ± 1.9 MPa, and for the 70/30 composites, it was 36.1 ± 1.2 MPa. The reason for the lowest shear strength values of the 70/30 composites was the low polymer content, which resulted in poor binding of the fiber filaments, and the polymer layer was too thin so it did not ensure the integrity of the composite. As a result, the formation of a weak interface was observed. Since the 60/40 composites showed the highest IPSS values, this composition was used to determine the influence of the CF surface modification on ILSS values. Additionally, the fiber to polymer ratio of 60/40 was used for all the modified fiber reinforced composites.

The IPSS results of the modified CF reinforced composites showed that the selected regimes of surface modification could significantly increase the shear strength of PSU-based composites. We found that thermal oxidation of the CF in the temperature range of 300–500 °C for 30 min allowed for an increase in shear strength up to 60–67.3 MPa (Figure 6b) (TO 300, 400, 500 designations mean the PSU based composites reinforced with CF after thermal oxidation at 300, 400, 500 °C, respectively). The increase in shear strength of the composites was due to the fact that, as shown by XPS analysis, the modification of the CF surface resulted in the formation of new functional groups on the fiber surface. Increase in the number of hydroxyl (–OH), carbonyl (–C=O), and carboxyl (–COOH) functional groups resulted in the chemical bonds between the filler and the polymer matrix formation, which resulted in the formation of a strong interfacial interaction in the composites. Favorable chemical and physical interaction between modified CF and PSU during the preparation of the composites https://www.sciencedirect.com/science/article/pii/S1359835X18303543-b0180 leads to the interfacial adhesion improvement [59,60]. As will be shown later, the observed improvement in interfacial interaction also resulted in a noticeable improvement in the mechanical properties of the composites reinforced with modified fibers.

Further demonstration of the CF surface modification positive effect on the interfacial interaction in the composites can be realized by SEM investigation of the fracture morphologies of the composites after tensile tests. Structural analysis of the composites reinforced with initial CF and after surface modification showed that the selected regimes could have a significant impact on the state of the interfaces, and, therefore, on the fracture mechanism.

**Figure 7:**
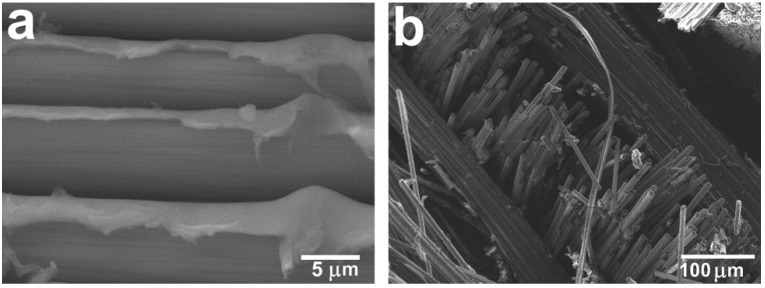
The structure of the CF/PSU composites (60/40 wt %) reinforced with the initial carbon fibers after tensile tests: (**a**) high-magnification image of the interfiber space; (**b**) low-magnification image of the fractured surface [43].

It was shown that the fiber–matrix interaction was significantly improved in the case of using CF after TO. As can be seen from Figure 7, the method applied for composites allowed us to achieve good impregnation of the fabrics with polysulfone due to the low-viscosity polymer solution used. The polymer penetrated not only between the individual fabric threads, but also impregnated the threads, penetrating between the CF individual filaments (Figure 7a). However, the composites reinforced with the initial CFs showed poor fiber–matrix interaction due to the inertness of the CF surface. In this case, mechanical interlocking between the PSU and CF was mainly realized. In this case, the CF surface remains clean and smooth; almost no polymer traces were observed on the CF surface, which means that during the mechanical tests, an intense destruction of the fiber–polymer interface occurs. Figure 7b shows that a number of fibers were pulled out from the PSU matrix, which was caused by the interface failure and suggests poor interfacial adhesion.

**Figure 8:**
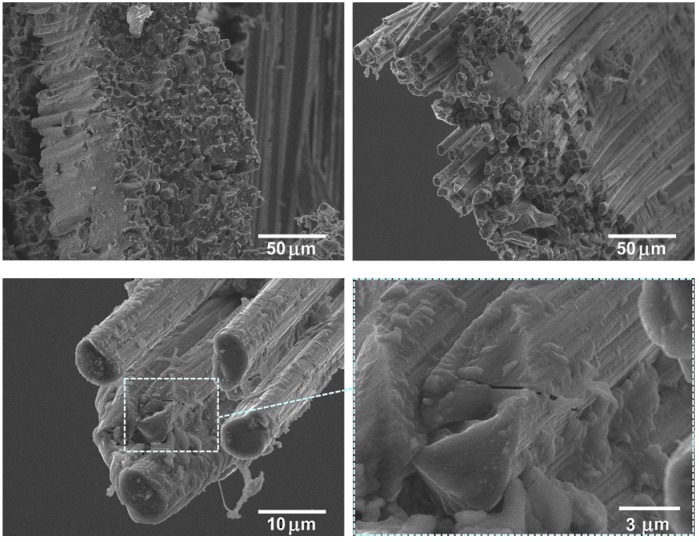
The structure of the carbon fiber/polysulfone composites (60/40 wt %) reinforced with surface modified carbon fibers (TO 500 °C, 30 min) after the tensile tests.

Comparatively, the CF surface modification significantly changed the composites’ fracture surface morphology. Figure 8 shows good fiber–matrix adhesion in the case of the composite containing thermally modified CFs rather than for composites reinforced with untreated CFs. Even after the mechanical tests, a significant amount of fragmented PSU adhered to the CF surface still remained. There was almost no CF pulled out from the PSU matrix and the interface debonding was reduced. In this case, the composites’ destruction proceeded with an equal probability both through the polymer matrix and the fibers, which is shown by the large quantity of destroyed CF ends located in the same level with the broken PSU surface of the polymer as well as presented in Figure 8 on high resolution image cracks directly in the CF. Formation of such a strong interface provides a good transfer of stress from the matrix material onto the CF, which is accompanied by a significant increase in the general mechanical properties of the composite.

### 3.4. Mechanical Tests of CF/PSU Composites

**Figure 9:**
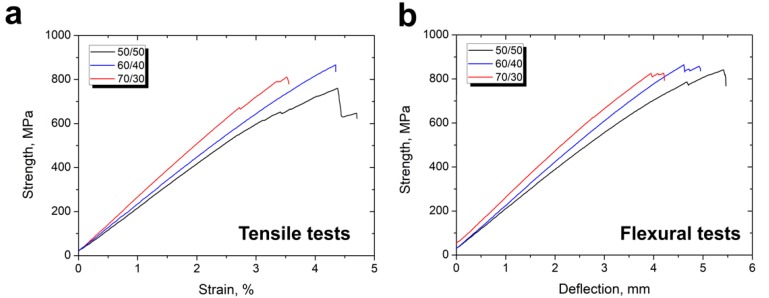
Typical stress–strain curves of the CF/PSU composites (**a**) during tensile and (**b**) flexural tests depending on the fiber content (initial fibers).

To further evaluate the mechanical properties of the CF/PSU composites, tensile and three-point flexural tests were performed using a universal testing machine. As known, reinforcement content is one of the main factors that affects the mechanical properties of fiber reinforced polymer composites. Fiber content strongly affect the stiffness, strength, and conductivity as well as other performance composite properties. Figure 9 shows typical stress–strain curves for the initial fiber reinforced PSU based composites during tensile and flexural tests depending on fiber content. The obtained curves were nearly linear, and the strain rate mainly depended on the fiber content. It can be seen that higher fiber content resulted in a lower strain rate that was caused by the fact that the carbon fibers had a much higher stiffness and lower plasticity compared with the polymer matrix.

**Figure 10:**
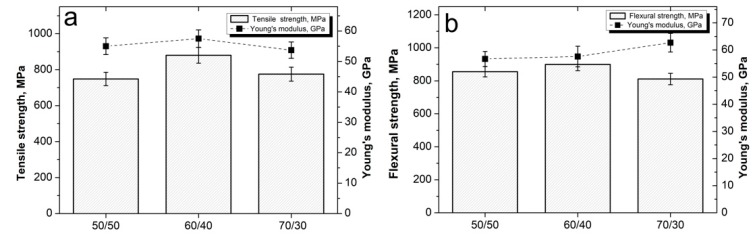
(**a**) Tensile and (**b**) flexural properties of the CF/PSU composites with various fiber to polymer ratios.

Figure 10a presents the tensile test results for composites with various fiber content. The minimum values of 748 ± 32 MPa and 55 ± 2.1 GPa for the ultimate tensile strength and Young’s modulus, respectively, was found for the composites with a fiber to polymer ratio of 50/50. Increase in the fiber content up to 60 wt % resulted in the increase in the tensile strength of the composites up to 880 ± 44 MPa and Young’s modulus up to 57.5 ± 2.2 GPa. An increase in the fiber content to 70% resulted in a decrease in the composite mechanical properties, the tensile strength decreased to 775 ± 3 6 MPa, and the Young’s modulus decreased to 53.7 ± 2.3 GPa.

Figure 10b shows the results of the flexural tests. It can be seen that the minimum value of the flexural strength (855 ± 31 MPa) was observed for the 50/50 composites. Increase in the fiber content up to 60% resulted in an increase in the strength of the composites up to 899 ± 27 MPa; a further increase in the CF content up to 70% resulted in a decrease in strength to 811 ± 28 MPa. The Young’s modulus in this case tended to increase from a value of 56.7 ± 1.7 GPa for a 50/50 composite up to 62.7 ± 2.4 GPa for a 70/30 composite.

We can summarize that at low CF content, the strength of the composites was rather low because of the significant content of the “weak” PSU phase. Increase in the CF content resulted in an increase in fiber packing density, while a decrease in the polymer interlayer between the individual fibers occurred. A decrease in the strength with a further increase in the CF content proceeded because the matrix content was too low to bind the CF and distribute the applied load to CF, in this case.

**Figure 11:**
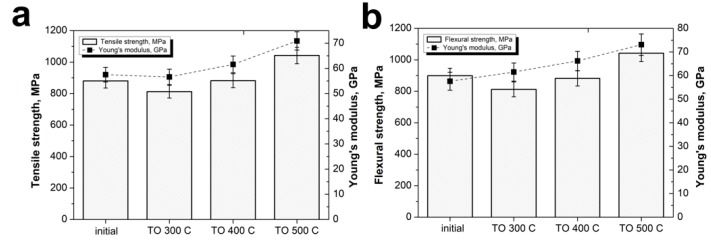
(**a**) Tensile and (**b**) flexural properties of the CF/PSU composites (60/40 wt %) reinforced with fiber after various surface modifications.

As above-mentioned, the mechanical properties of composites mainly depend on the fiber–matrix interfacial interaction. Tensile and flexural tests of the composites reinforced with CFs after TO were performed. In this case, composites with an optimum fiber content of 60 wt % that showed the maximum values of the strength-elastic properties were used. Figure 11 shows that the CF surface modification significantly affected the composites’ mechanical characteristics.

The maximum ultimate strength and Young’s modulus values were obtained at the TO temperature of 500 °C. In this case, the tensile strength and Young’s modulus of the composites increased from the initial 880 ± 44 MPa and 57.5 ± 2.2 GPa up to 1047 ± 2.8 MPa and 70.9 ± 2.6 GPa, respectively. The same behavior after the flexural tests was observed: from the initial 899 ± 27 MPa and 57.6 ± 2.8 GPa, the flexural strength and flexural modulus increase up to 1042 ±3.2 MPa and 73.1 ± 3.5 GPa, respectively. The observed increase in mechanical properties, due to the good CF/PSU interfacial interaction, and a stronger interface for the composites reinforced with modified fibers in comparison with initial fiber reinforced composites, allows for an improvement in the mechanical properties of the composites.

### 3.5. Dynamic Mechanical Analysis

**Figure 12:**
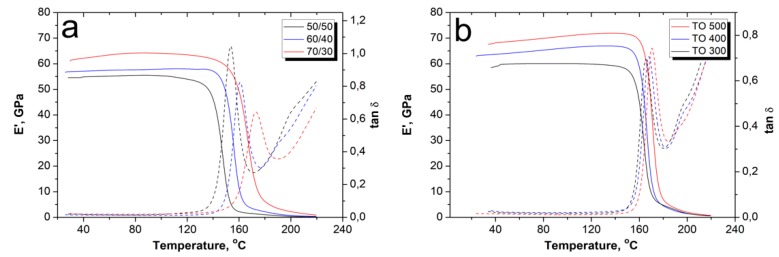
The DMA results of the CF/PSU composites (60/40 wt %) depending on the (**a**) initial fiber content and the (**b**) fibers’ surface modification regime.

The effect of the TO of CF on the composites’ dynamic mechanical response was studied by DMA. This method of thermal analysis, which allows the mechanical properties of a material to be measured during its periodic deformation, makes it possible to determine the thermal behavior of a material under cyclic loads. Figure 12 shows the storage modulus and tan δ of the CF/PSU composites, which was plotted as a function of temperature. Storage modulus indicates a measure of the elastic response of the composite and tan δ (=E″/E′) is equal to the ratio of the loss modulus (E″) to the storage modulus (E′), thus representing the damping capacity of a material. It was shown that for all samples, the values of E′ decreased gradually with increasing temperature, followed by a sharp drop after the Tg region (Figure 12a).

The behavior of the storage modulus values at room temperature of the obtained composites with different fiber to polymer ratios was similar to the behavior during the flexural tests (see Figure 9). The minimum values of the E′ were observed for the 50/50 composites and the highest values were observed for the 60/40 composites. Up to temperatures of 130–140 °C, the storage modulus of the composites underwent only slight changes, however, above this temperature, the E′ values began to decrease. It can be seen that the temperature of the beginning of the E′ decrease and the temperature to which the samples retain their stability depend on the fiber content. The lowest thermal stability was demonstrated by the 50/50 composites, which lost their stability at temperatures more than 10 °C less than that for the 60/40. The 70/30 composites showed the best thermal stability. The observed decrease in storage modulus was due to the softening of the polymer matrix due to glass transition. The softening of the PSU can be judged by the behavior of tan δ, as shown in Figure 12a. Usually, the maximum of the tan δ peak relates to the relaxation processes of the polymer matrix such as glass transition (α-transition), melting, or other. In our case, the tan δ peaks were affected by the fiber to polymer ratio, and were 155, 161, and 172 °C, for 50/50, 60/40, and 70/30 composites, respectively. Consequently, an increase in the fiber content resulted in a slight increase in the thermal stability of the composites, which was caused both by a decrease in the mobility of the macromolecular segments of the polymer matrix and by a decrease in the volume of the polymer matrix, which is prone to softening at temperatures close to the glass transition temperature. As a result of softening of the polymer matrix, it was no longer able to transfer an external load to the reinforcing fibers, which was accompanied by a sharp drop in the mechanical properties of the composites.

It was shown that the CF surface modification allowed for an increase in the storage modulus of the composites and shifted the temperature of the tan δ peak toward higher temperatures. In Figure 12b, it can be seen that the higher TO temperature resulted in the higher tan δ peak temperature, which corresponded to temperatures of 165, 167, and 170 °C for the composites reinforced with fibers after TO at 300, 400, and 500 °C, respectively. This is due to the fact that reinforcement with modified fibers reduces the free volume and decreases the polymer chains’ mobility because of strong interfacial interaction between the CF and PSU. An increase in the interfacial interaction between the fibers and the matrix means that the mobility of the chains decreases even more, which results in higher thermal stability temperatures of the tan δ peak.

## 4. Conclusions

The solution impregnation method was used to form PSU based composites reinforced with twill wave CF fabrics. The elaborated technique allowed us to achieve homogeneous impregnation of CFs with the polymer and to further remove the solvent from the composite almost completely. Low viscosity of the impregnation solution resulted in polymer penetration even between the individual CF filaments. To improve the adhesion between CF and PSU, the surface of the fabrics was modified by thermal oxidation in an air atmosphere. It was shown that the surface modification by heating at a temperature to 500 °C for 30 min significantly changes the functional composition of the CF surface. Hydroxyl –C–OH, ethereal –C–O–C–, carboxyl COOH–, carbonyl –C=O, and epoxy groups form on the CF surface. Shear strength tests and SEM analysis of the fracture surface confirmed that surface modification resulted in a significant increase in the adhesion between the CF and polymer matrix. The in-plane shear strength magnitude for composites reinforced with modified CF fabrics was found to be more than 1.5 times higher than for composites containing unmodified CFs. Fracture analysis showed that in the case of unmodified CF, destruction proceeded on the boundary between the CFs and PSU. In the case of modified CF, the destruction of composites occurred both through the polymer matrix and CF body.

Mechanical tests in the tensile and flexural modes were carried out for the obtained composites. Carbon fiber content affects the composites’ mechanical properties. At low CF content, the composite strength was not that high due to the significant content of the “weak” polymer phase. Increase in the fiber content provided an increase in the fiber packing density, while a decrease in the polymer interlayer between the individual fibers occurred. Decrease in the strength at a further increase in the CF content proceeded because the matrix content was too small to bind the CF and distribute the applied load to the CFs in this case. Surface modification of CF also led to an increase in the tensile and flexural properties of the composites. The maximum ultimate strength and Young’s modulus values were found in the case of the CF TO temperature value of 500 °C. In this case, the tensile strength and Young’s modulus of the composites increased from the initial 880 ± 44 MPa and 57.5 ± 2.2 GPa up to 1047 ± 28 MPa and 70.9 ± 2.6 GPa, respectively. The same behavior after the flexural tests was observed: from the initial 899 ± 27 MPa and 57.6 ± 2.8 GPa, the flexural strength and flexural modulus increased up to 1042 ± 32 MPa and 73.1 ± 3.5 GPa, respectively. Dynamic mechanical analysis showed that the investigated composites were stable up to the temperatures of 130–140 °C, and that an increase in the CF content was accompanied by an increase in the thermal stability of the composites. Surface modification of CFs also resulted in an increase in the composites’ thermal stability due to the decrease in the polymer chains mobility, which was caused by good interfacial interaction between the CFs and PSU.

## Figures and Tables

**Table 1:** Concentration of different elements in atomic percent obtained from the x-ray photoelectron spectroscopy results for the carbon fiberssurface before and after thermal oxidation (TO).

Sample	Concentration, at. %							
	C	O	N	Si	S	Na	Cl	F
initial	77.5	18.0	3.0	0.7	0.3	0.3	0.3	-
TO 300 °C	79.0	18.1	0.4	2.3		0.1		0.1
TO 500 °C	82.5	7.5	8.5	0.3	-	-		1.0

**Table 2:** Binding energies (E_B_) obtained from the high resolution spectra of the initial and modified carbon fibers [43].

Sample		C					O		N		
		1	2	3	4	5	1	2	1	2	3
**Initial**	E_B_, eV	285.0	286.5	289.5	-	-	531.7	532.9	400.3		
	%	65	32	3	-	-	15	85	100		
**TO 300 °C**	E_B_, eV	285.0	286.5	289.5	-	291.5	531.3	533.5	400.3		
	%	69	23	4	-	4	10	90	100		
**TO 500 °C**	E_B_, eV	284.7	286.1	287.5	288.6	291.4	531.3	533.4	398.7	400.55	403.5
	%	80	9	3	2	6	50	50	40	50	10
